# Dynamic Changes in Coagulation Function in Patients With Pneumonia Under Admission and Non-admission Treatment

**DOI:** 10.3389/fmed.2021.626384

**Published:** 2021-05-24

**Authors:** Jiasheng Xu, Yongmei Zhang, Yiran Li, Kaili Liao, Xiong Zeng, Xiande Zeng, Rui Meng, Weimin Zhou, Kai Wang, Yuanqi Gong, Fuzhou Hua, Jianjun Xu, Jiehua Qiu

**Affiliations:** ^1^Department of Vascular Surgery, The Second Affiliated Hospital of Nanchang University, Nanchang, China; ^2^Department of Clinical Laboratory, The Second Affiliated Hospital of Nanchang University, Nanchang, China; ^3^Queen Mary College of Nanchang University, Nanchang, China; ^4^The Center of Oncology, Wuhan Union Hospital, Huazhong University of Science and Technology, Wuhan, China; ^5^Department of Hepatobiliary and Pancreatic Surgery, The Second Affiliated Hospital of Nanchang University, Nanchang, China; ^6^The Second Affiliated Hospital of Nanchang University Aid Hubei Province to Against the Epidemic of New Coronavirus National Medical Team, Nanchang, China; ^7^Intensive Care Unit, The Second Affiliated Hospital of Nanchang University, Nanchang, China; ^8^Department of Anesthesiology, The Second Affiliated Hospital of Nanchang University, Nanchang, China; ^9^Department of Cardiac Surgery, The Second Affiliated Hospital of Nanchang University, Nanchang, China

**Keywords:** coagulation function, pneumonia, COVID 19, the dynamic changes, treatment

## Abstract

**Objective:** We aimed to explore the dynamic changes in coagulation function and the effect of age on coagulation function in patients with pneumonia under admission and non-admission treatment.

**Methods:** We included 178 confirmed adult inpatients with COVID-19 from Wuhan Union Hospital Affiliated to Huazhong University of Science and Technology (Wuhan, China). Patients were classified into common types, and all were cured and discharged after hospitalization. We recorded the time of the first clinical symptoms of the patients and performed blood coagulation tests at the time of admission and after admission. In total, eight factors (TT, FIB, INR, APTT, PT, DD, ATIII, and FDP) were analyzed. Patients were classified into four groups according to the time from the first symptom onset to hospital admission for comparative analysis. The patients who were admitted within 2 weeks of disease onset were analyzed for the dynamic changes in their blood coagulation tests. Further division into two groups, one group comprising patients admitted to the hospital within 2 weeks after the onset of disease and the other comprising patients admitted to the hospital 2 weeks after disease onset, was performed to form two groups based on whether the patient ages were over or under 55 years. Chi-square tests and *T* tests were used to explore the dynamic changes in coagulation function and the influence of age on the results of coagulation function tests.

**Results:** A total of 178 inpatients, 34 of whom underwent dynamic detection, were included in this analysis. We divided these patients into four groups according to the interval between the onset of COVID-19 pneumonia and the time to admission in the hospital: the 1–7 days (group 1), 8–14 days (group 2), 15–21 days (group 3), and >21-days (group 4). Eight factors all increased within 2 weeks after onset and gradually decreased to normal 2 weeks before the patient was admitted. The changes in coagulation function of patients admitted to the hospital were similar. After being admitted to the hospital, the most significant decreases among the eight factors were between week 2 and 3. There were distinct differences among the eight factors between people older than 55 years and those younger than 55 years. In the first 2 weeks after being admitted, the levels of the eight factors in patients >55 years were significantly higher than those in patients <55 years, and after another 2 weeks of treatment, the factor levels in both age groups returned to normal.

**Conclusion:** The eight factors all increased within 2 weeks after onset and gradually decreased to normal after 2 weeks regardless of treatment. Compared with patients younger than 55 years, patients older than 55 years have greater changes in their blood coagulation test values.

## Introduction

A novel coronavirus (2019-nCoV), first reported in Wuhan, China, in December 2019, led to an outbreak of pneumonia that spread across China rapidly and aroused enormous attention worldwide. Coronavirus disease 2019 (COVID-19) refers to 2019-nCoV-induced pneumonia. In COVID-19 patients, fever, dry cough, and dyspnea occur, and in more severe cases, acute respiratory distress syndrome (ARDS), septic shock, metabolic acidosis, bleeding, and coagulation dysfunction are observed (1). During the early stage of this pneumonia, symptoms of severe acute respiratory infection may develop, and acute respiratory distress syndrome, acute respiratory failure, and other serious complications occur rapidly in some patients. Until now, no available vaccines or clinically approved antiviral drugs have been used to treat these human coronavirus infections. Notably, postmortem autopsy found sulfuric acid in the body of COVID-19 patients, which indicates that the formation of thrombi was caused by a change in coagulation function, eventually resulting in the death of the patient.

Over time, there has been an increasing number of studies; however, there are few studies on the coagulation functional changes in COVID-19 patients. Most of the previous studies have focused on D-dimer and its importance in the prediction of COVID-19. However, the coagulation function dynamics of COVID-19 patients, particularly ordinary COVID-19 patients, are not reported.

The study's aim was to evaluate dynamic changes in coagulation function in ordinary patients with COVID-19 under admission and non-admission treatment.

## Methods

### Study Design

This is a retrospective study. Consecutive patients with ordinary COVID-19 admitted to Wuhan Union Hospital (Tumor Center) of Huazhong University of Science and Technology in Wuhan from February 12 to March 15, 2020, were retrospectively enrolled. All of the enrolled subjects had COVID-19, which was confirmed by detection of the RNA and specific IgG and IgM of 2019-nCoV and chest CT scans. All patients were cured and discharged after hospitalization. The study was approved by the Research Ethics Commission of the Second Affiliated Hospital of Nanchang University. The study followed the law of China, Declaration of World Medical Association (2), and TREND (improving the reporting quality of non-randomized evaluations of behavioral and public health interventions statement).

#### Inclusion Criteria

Patients meeting the following criteria were included in the study: (1) history of clear symptoms; (2) at least one positive result for RNA detection of 2019-nCoV; (3) positivity for IgG and IgM of 2019-nCoV and negativity for 2019-nCoV RNA before discharge; (4) presence of relative changes in the lung CT scan; (5) showing recovery from the disease and being discharged during the hospitalization period; and (6) aged between 18 to 85 years.

#### Exclusion Criteria

Patients were excluded from the study if they met any of the following criteria: (1) need for a tracheal cannula in the hospital; (2) chronic kidney failure or liver dysfunction; (3) comorbidity of low-extremity deep vein thrombosis (DVT); (4) another positive 2019-nCoV RNA test result after discharge; and (5) positive 2019-nCoV RNA test results but no relative signs on the chest CT scan.

### Data Sources and Data Collection

All patients were treated by the Second Affiliated Hospital of Nanchang University Aid Hubei Province against the Epidemic of COVID-19 National Medical Team and Tumor Center of Wuhan Union Hospital team. They were diagnosed with COVID-19 on the basis of WHO interim guidance. The patient medical history, which included the onset time of the first symptom(s), was recorded. Patients were classified into four groups based on the time from onset of the first symptom to hospitalization: 1–7 days (group 1), 8–14 days (group 2), 15–21 days (group 3), and over 21 days (group 4). The coagulation profiles of all patients were obtained when they were admitted to the hospital. In group 1, coagulation profile values were analyzed every week. Additionally, patients admitted to the hospital within 2 weeks or more than 2 weeks after the onset of symptoms were divided into subgroups based on age (55 years): group 12O (patients admitted to the hospital within 2 weeks after the onset of symptoms who were older 55 years), group 12Y (patients admitted to the hospital within 2 weeks after the onset of symptoms who were younger than 55 years), group 34O (patients admitted to the hospital more than 2 weeks after the onset of symptoms who were older than 55 years), and group 34Y (patients admitted to the hospital more than 2 weeks after the onset of symptoms who were younger than 55 years). Demographic, epidemiological, laboratory, clinical, treatment, and outcome data were extracted using a standardized data collection form from electronic medical records, which was a modified version of the WHO/International Severe Acute Respiratory and Emerging Infection Consortium case record form for severe acute respiratory infections. Routine blood tests included a coagulation profile, complete blood count, serum biochemical tests, myocardial enzymes, interleukins (ILs), serum ferritin, and procalcitonin. The coagulation profile included fibrinogen (FIB), fibrinogen degradation product (FDP), D-dimer (DD), thrombin time (TT), international normalized ratio (INR), activated partial thromboplastin time (APTT), prothrombin time (PT), and antithrombin III (ATIII). The coagulation indexes were analyzed weekly. The mean value was used if there was more than one coagulation profile test. All inpatients underwent chest CT scans. The frequency of the examination was determined by the treating physician. Laboratory confirmation of COVID-19 was performed by real-time RT-PCR of 2019-nCoV RNA from throat swab specimens. After remission of clinical symptoms (including fever, cough, and dyspnea), throat swabs were collected every other day for 2019-nCoV PCR re-examination at least two times.

The severity of COVID-19 was defined in accordance with the Chinese management guideline for COVID-19 (version 6.0). Fever was defined as an axillary temperature of at least 37.3°C. The discharge criteria were as follows: no fever for at least 3 days, significant improvement in chest CT in both lungs, relief of clinical respiratory symptoms, and negative SARS-CoV-2 RNA detected in at least two throat swabs 24 h apart (3–6). Coagulopathy was defined as a prothrombin time of 3 s prolongation or an activated partial thromboplastin time of 5 s extension.

### Statistical Analysis

Continuous and categorical variables are presented as the median and standard deviation (SD). We used the chi-square test and *T* test to explore the dynamic changes in coagulation function and the influence of age on the results of the coagulation function test. Multivariate analysis of variance was used to study the changes in the eight related indexes of coagulation function in patients with COVID-19.

### Results

In total, 178 patients, 94 males and 84 females, were included in the final analysis, and the mean age was 57.2 ± 15.3 years. There were 41, 44, 38, and 55 patients in group 1, group 2, group 3, and group 4, respectively. Nearly half of the patients had comorbidities. Hypertension was the most common comorbidity, followed by diabetes and coronary heart disease. The comparison of patient characteristics among the four groups is shown in Table 1. In group 1, 34 patients showed weekly gains in the coagulation index after four assessments. Additionally, there were 55, 29, 53, and 41 patients in group 12O, group 12Y, group 34O, and group 34Y, respectively.

**Table 1:** The characteristics of patients in this study.

**Variables**	**1–7 day**	**8–14 day**	**15–21 day**	**>21 day**
Number of patients	41	44	38	55
Gender (M/F)	16/25	21/23	21/17	11/19
Age (Y)	60.1 ± 16.8	63.4 ± 12.9	56.5 ± 15.2	52.9 ± 16.6
**Comorbidity**				
Hypertension	14	17	18	12
Coronary disease	5	3	1	1
Other heart disease	2	1	1	1
Diabetes	1	2	3	4
Respiratory disease	2	2	0	3
DVT	2	2	0	0
Others	6	5	3	6

**Figure 1:**
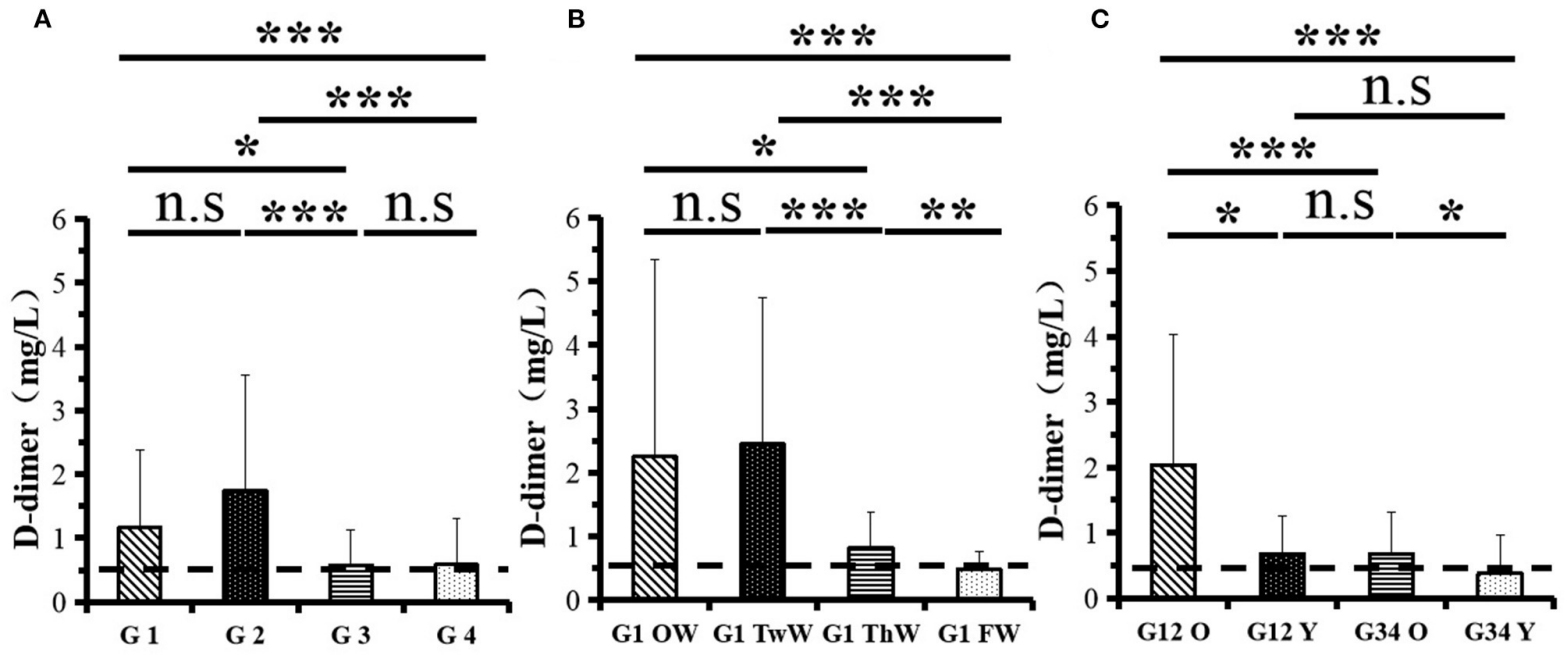
D-dimer (DD).

**Figure 2:**
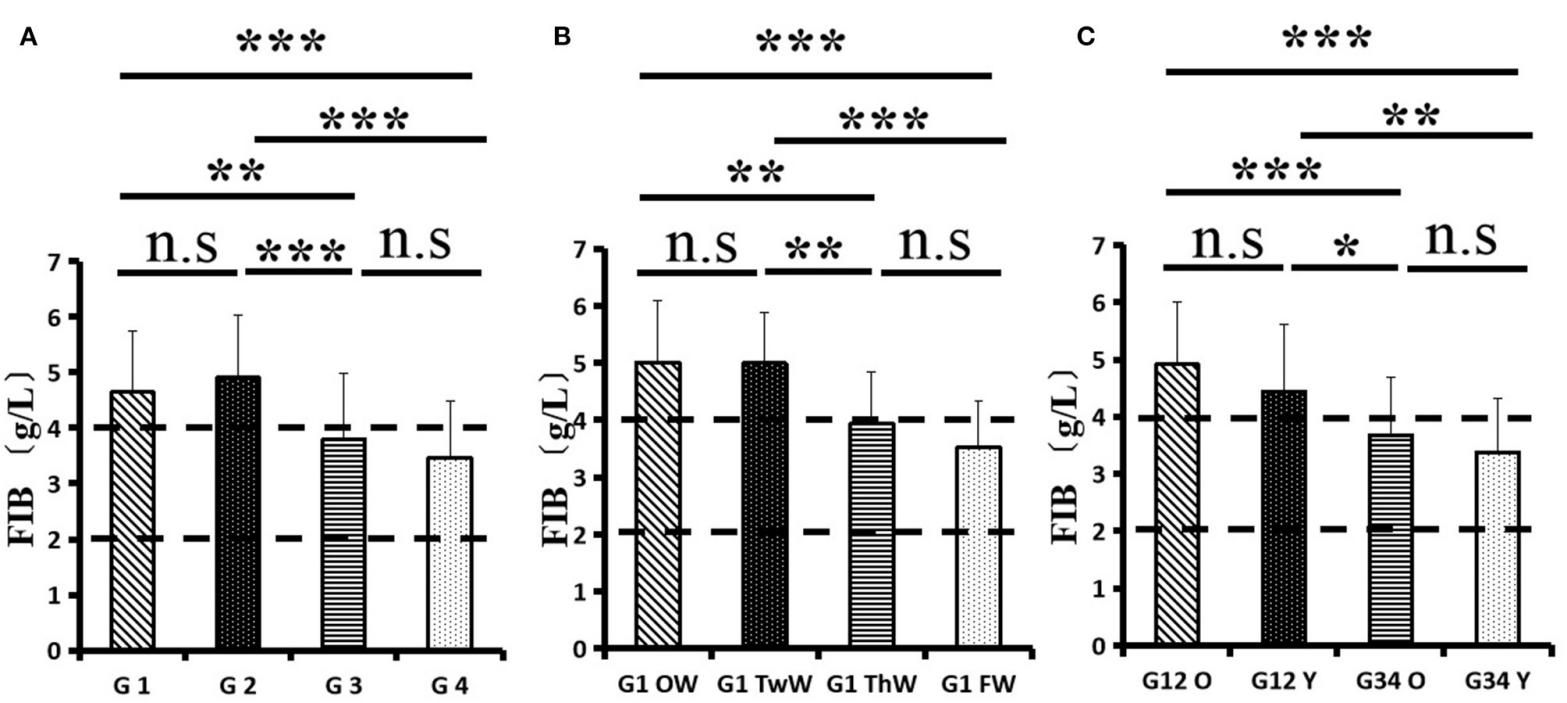
Fibrinogen (FIB).

**Figure 3:**
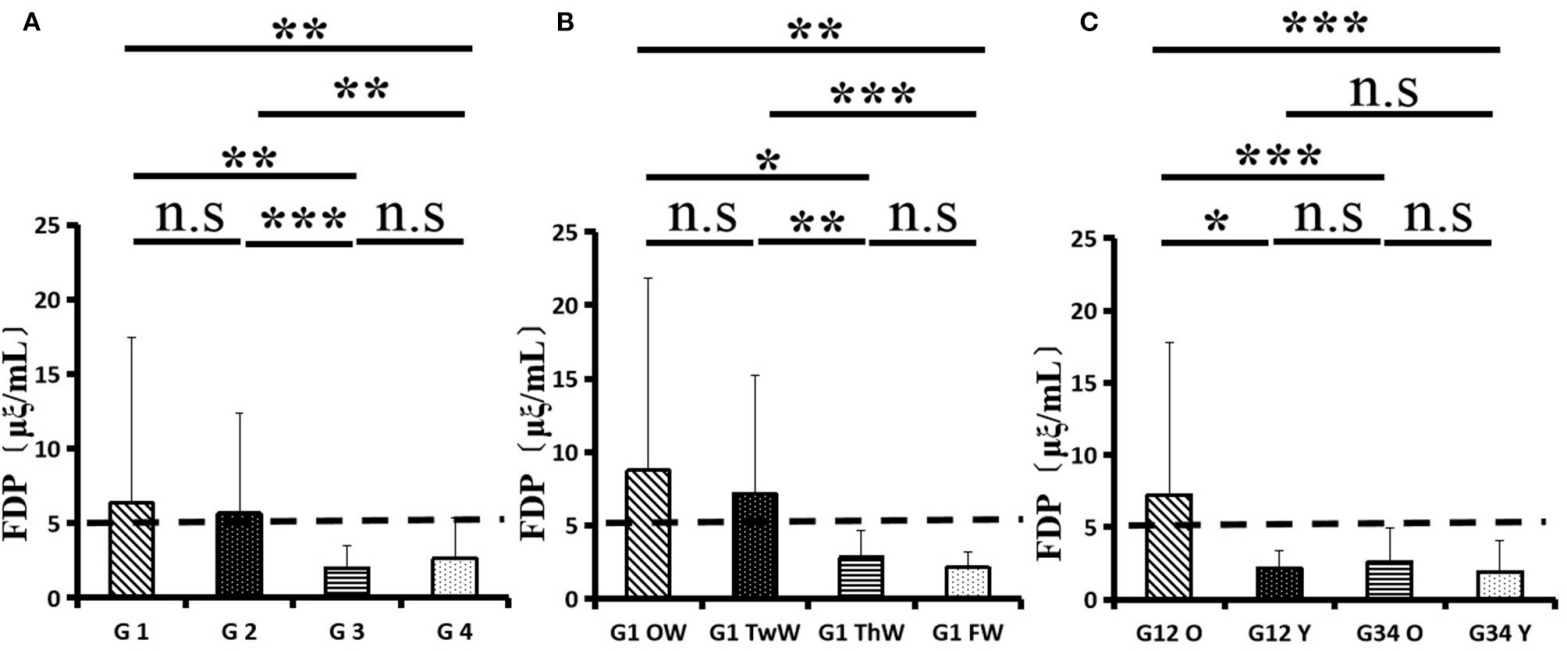
Fibrinogen degradation product (FDP).

**Figure 4:**
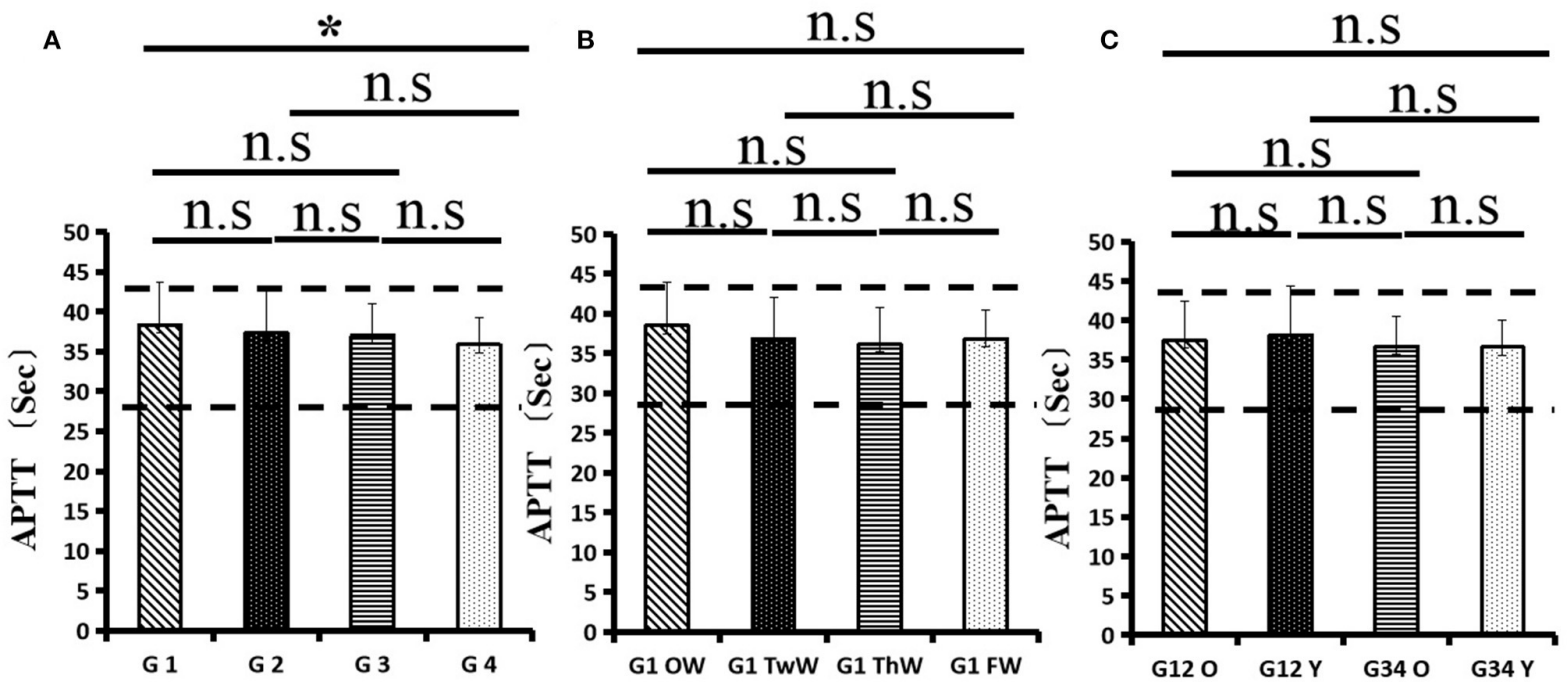
Activated partial thromboplastin time (APTT).

**Figure 5:**
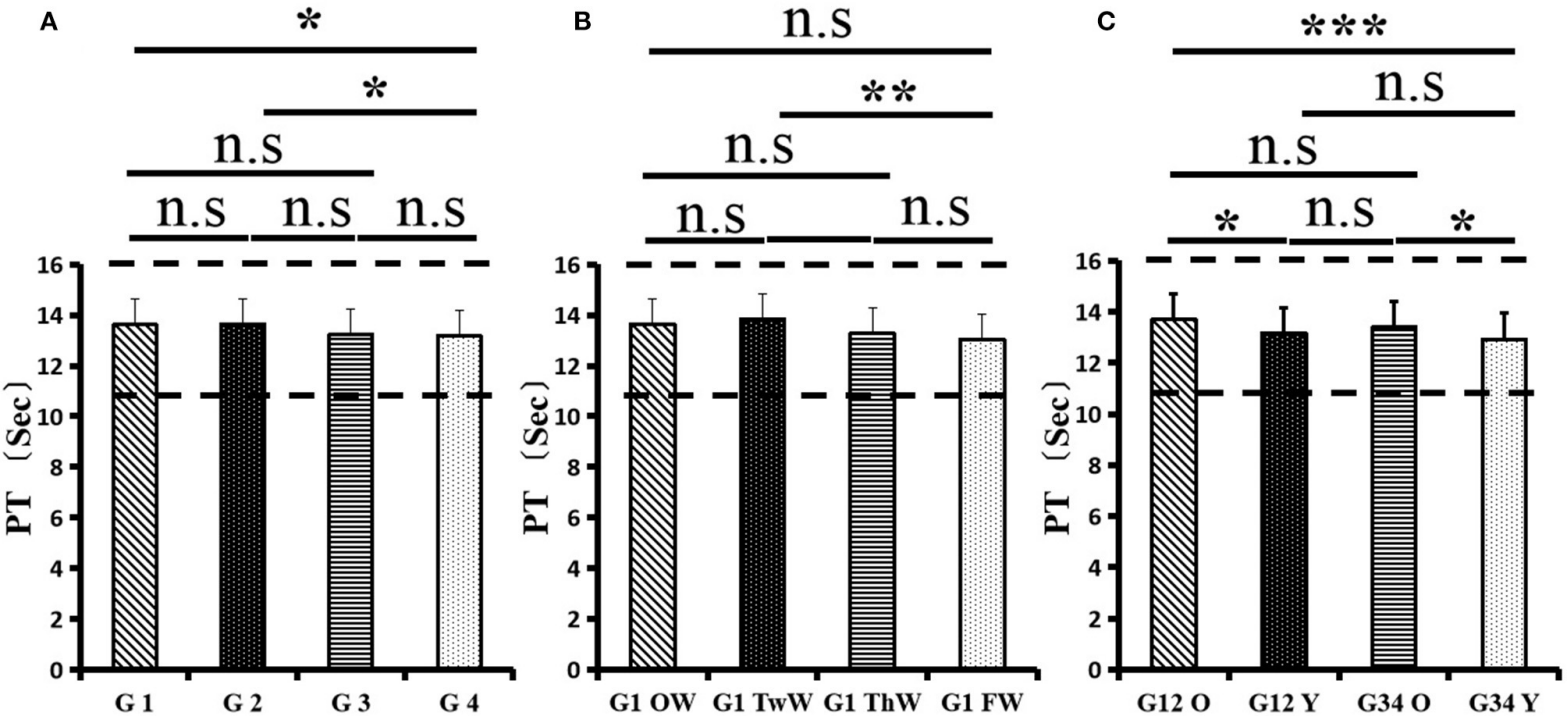
Prothrombin time (PT).

**Figure 6:**
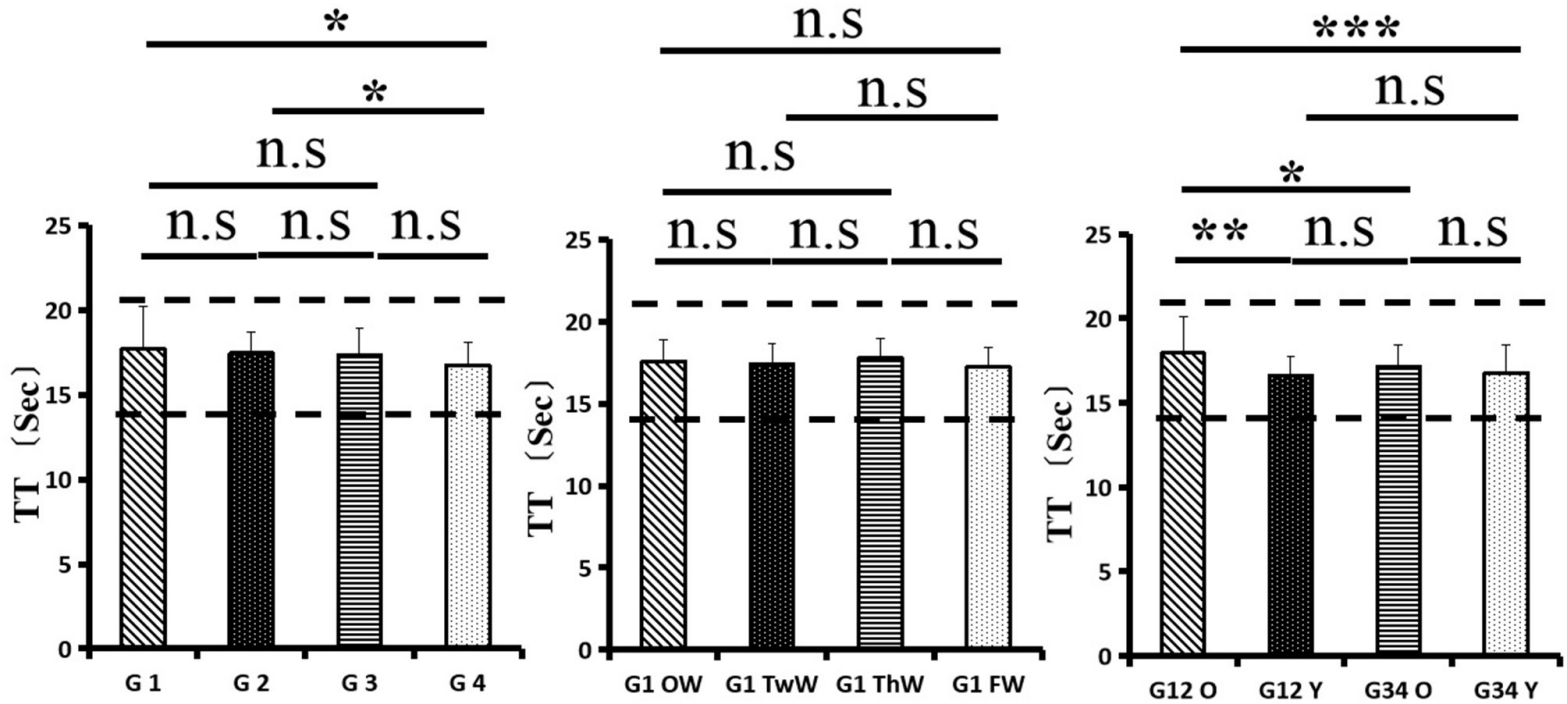
Thrombin time (TT).

**Figure 7:**
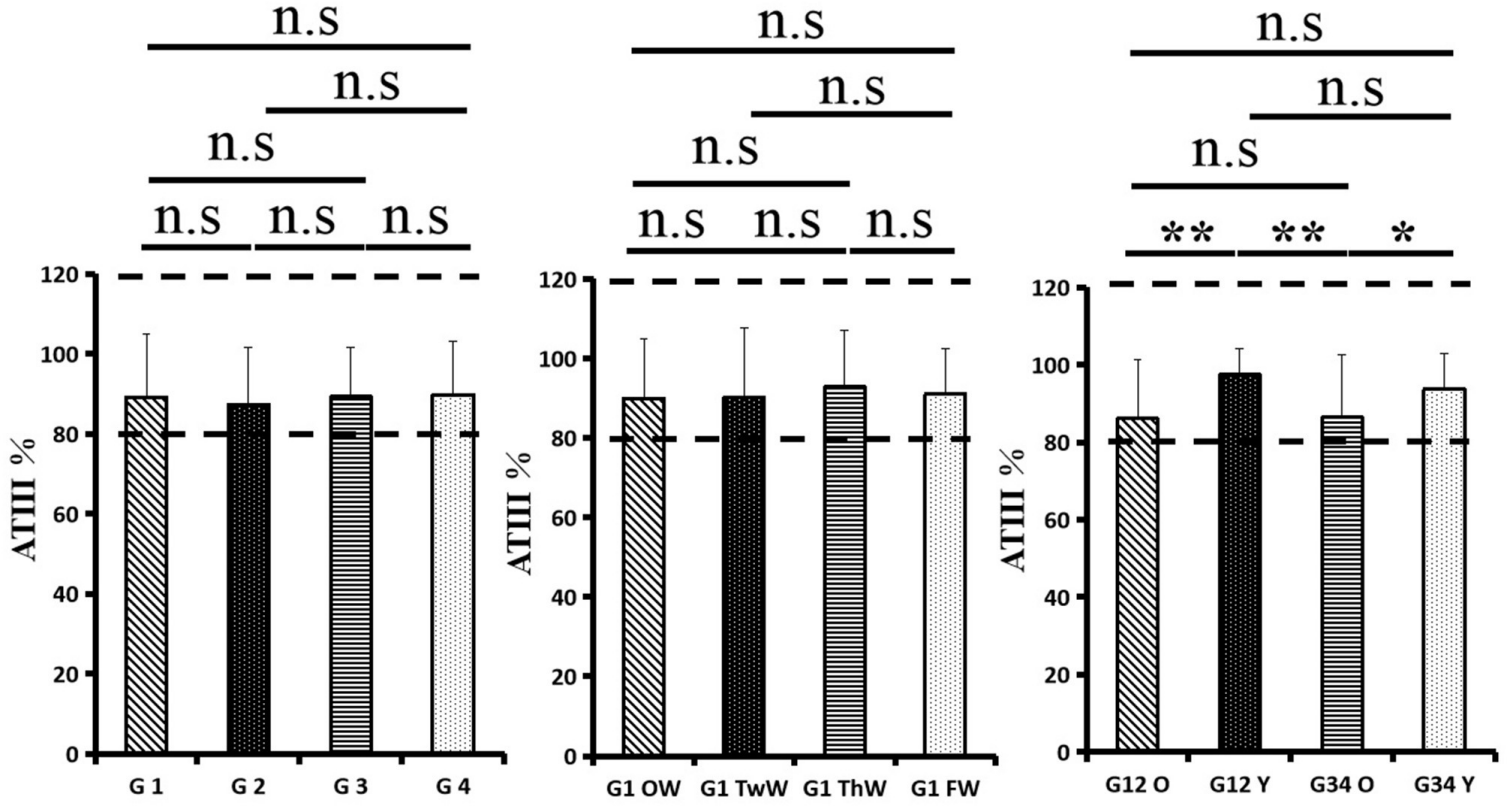
Antithrombin III (ATIII).

**Figure 8:**
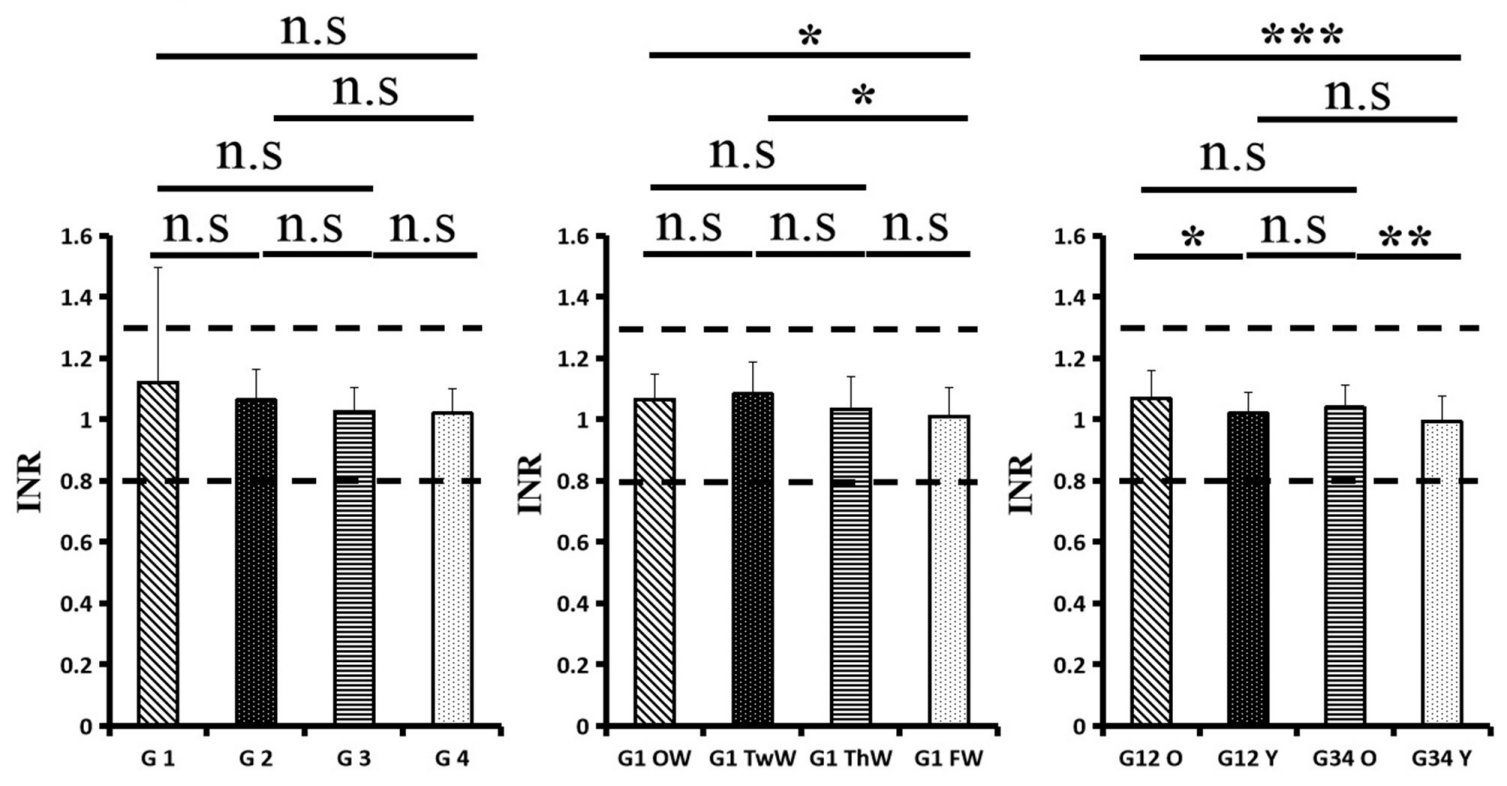
International normalized ratio (INR).

Changes in eight factors that indicate coagulation function, namely, TT, FIB, INR, APTT, PT, DD, ATIII, and FDP, were analyzed. In the analysis of the data from the 1-week group, 2-week group, 3-week group, and 4-week group, the eight factors all initially increased and then decreased to a lower level. In group 1, the DD level was 1.16 ± 1.22 (units), APTT level was 38.30 ± 5.38, PT level was 13.62 ±0.87, ATIII level was 89.19 ± 15.92, FDP level was 6.36 ± 11.10, INR level was 1.12 ± 0.37, FIB level was 4.65 ± 1.11, and TT level was 17.70 ± 2.52 (Figures 1A–8A). In group 2, the DD level was 1.75 ± 1.81 (units), APTT level was 37.31 ± 5.42, PT level was 13.62 ± 0.99, ATIII level was 87.39 ± 14.12, FDP level was 5.67 ± 6.70, INR level was 1.06 ± 0.10, FIB level was 4.91 ± 1.13, and TT level was 17.44 ± 1.22 (Figures 1B–8B). In group 3, the DD level was 0.57 ± 0.55 (units), APTT level was 37.00 ± 3.99, PT level was 13.25 ± 0.81, ATIII level was 89.29 ± 12.41, FDP level was 2.01 ± 1.46, INR level was 1.03 ± 0.08, FIB level was 3.80 ± 1.18, and TT level was 17.35 ± 1.54 (Figures 1C–8C). In group 4, the DD level was 0.58 ± 0.71 (units), APTT level was 89.83 ± 13.43, PT level was 13.19 ± 0.83, ATIII level was 89.29 ± 12.41, FDP level was 2.64 ± 2.69, INR level was 1.02 ± 0.08, FIB level was 3.47 ± 1.01, and TT level was 16.73 ± 1.33 (Figures 1A–8A). FDP (the *p*-value between group 1 and group 2 was 0.85) and DD (the *p*-value between group 1 and group 2 was 0.11) had the largest changes.

After the admission of the 178 patients to the hospital, the eight factors were measured dynamically and could be used to analyze continuous changes in different disease stages. The overall trend was decreased to the normal level after treatment. After 1 week of treatment, the DD level was 2.25 ± 3.10, APTT level was 38.5 ± 5.42, PT level was 13.64 ± 0.85, ATIII level was 89.92 ± 14.86, FDP level was 8.74 ± 13.12, INR level was 1.07 ± 0.08, FIB level was 5.01 ± 1.07, and TT level was 17.55 ± 1.30. After 2 weeks of treatment, the DD level was 2.45 ± 2.30, APTT level was 36.87 ± 5.19, PT level was 13.84 ± 0.99, ATIII level was 90.31 ± 17.44, FDP level was 7.15 ± 8.09, INR level was 1.08 ± 0.10, FIB level was 4.99 ± 0.89, and TT level was 17.43 ± 1.19. After 3 weeks of treatment, the DD level was 0.83 ± 0.56 and continuously decreased to 0.48 ± 0.29 by week 4, the APTT level was 36.14 ± 4.59 and increased slightly to 36.83 ± 3.57 by week 4, the PT level was 13.84 ± 0.99 and decreased slightly to 13.04 ± 0.86 by week 4, the ATIII level was 92.86 ± 14.15 and decreased to 91.03 ± 11.52 after another week of treatment, the FDP level was 2.83 ± 1.86 and continuously decreased to 2.12 ± 1.10 by week 4, the INR level was 1.03 ± 0.11 and decreased slightly to 1.01 ± 0.09 in week 4, the FIB level was 3.94 ± 0.91 and decreased to 3.53 ± 0.80, and the TT level was 17.76 ± 1.20 and decreased slightly to 17.22 ± 1.20 after another week treatment. The most significant decrease was between week 2 and week 3 (*p* < 0.001).

It was found that old age might be connected with an increase in mortality because most old populations have low immune function and comorbidities such as hypertension, diabetes, and coronary heart disease. However, the exact age at which the patient was more likely to be infected is currently unknown. In this study, we noticed that the eigth factors mentioned above had distinct differences between people older than 55 years and those younger than 55 years. In the first 2 weeks, the DD level in patients >55 years was significantly higher than that in patients <55 years (*p* value was 0.03), and after another 2 weeks of treatment, the DD level in both age groups returned to normal. The FDP level had a similar changing pattern; the FDP level was 7.24 ± 10.55 during the first 2 weeks of treatment among patients older than 55 years and was much higher than that in patients <55 years (2.15 ± 1.23). After another 2 weeks of treatment, the FDP levels of both age groups returned to normal. The PT level in older patients decreased from 13.7 ± 0.87 to 13.39 ± 0.75 after another 2 weeks of treatment. Compared with older patients, young patients had a lower PT level, and after 2 weeks of treatment, it decreased from 13.15 ± 0.74 to 12.93 ± 0.85. The INR level also had a similar changing pattern; it decreased from 13.7 ± 0.87 to 13.39 ± 0.75 after another 2 weeks of treatment in patients older than 55 years, and young patients had a lower INR level than older patients. After 2 weeks of treatment, it decreased from 1.02 ± 0.07 to 0.99 ± 0.84. Similar to the FIB level change, the older patients' FIB level decreased from 4.91 ± 1.09 to 3.68 ± 1.00, and the younger patients' FIB level decreased from 4.44 ± 1.17 to 3.38 ± 0.93.

## Discussion

This is an extended descriptive study on the characteristics of coagulation function changes in 2019-nCoV patients, which included data on more than 150 patients with 2019-nCoV, all of whom presented with mild clinical symptoms and were cured and discharged after hospitalization. 2019-nCoV-infected patients can present with a variety of secondary hematological, immunological and biochemical pathophysiological changes, with severe acute interstitial infiltration, leakage inflammation, and other lesions, combined with secondary hyperfibrinogenemia, in patient lung tissues (7). Due to the release of various inflammatory factors and coagulant substances during infection, blood is in a hypercoagulable state. Together with hypoxia and acidosis, hypercoagulability can cause pulmonary microvasoconstriction and blood retardation, and the progression of microthrombosis can aggravate pulmonary respiratory disorders (8). In mild cases, there may be no formation of blood clots or formation of small blood clots, which may be dissolved by the fibrinolytic system quickly.

In this study, we used 7 days as the cutoff point and classified patients with onset-to-admission times within 1–7 days into the early group and those with onset-to-admission times above 7 days into the advanced group. The serum DD, FIB, and IL-6 concentration values of the two groups were compared at the time of admission to observe whether there was a difference. DD, FIB, and IL-6 levels in patients with mild 2019-nCoV pneumonia were higher than those in the normal physical examination, and the factors in these patients on the 7^th^ day before being admitted to the hospital were significantly higher than those on the 7^th^ day after they were treated. This study found that old age may be associated with increased mortality because most of the older population has low immune function and is associated with complications such as high blood pressure, diabetes, and coronary heart disease. However, the exact age at which people were most likely to be infected is unknown. In this study, we noted that there were significant differences in the above eight factors between people over 55 years old and those under 55 years old. In the 2019-nCoV cases analyzed in this paper, the plasma FIB concentration increased significantly in the acute onset phase, plasma DD was obviously elevated, FIB increased mildly, and TT, INR, APTT, PT, and ATIII were still under normal range. All of these patients recovered and were discharged after treatment, and the factors gradually returned to normal. Analyzing abnormal changes in blood coagulation function in 2019-nCoV patients is important for the further understanding and treatment of 2019-nCoV in the future.

FIB is not only the most abundant macromolecular coagulation factor in plasma but also an acute-phase protein (9). The significant increase in plasma FIB concentration in 2019-nCoV patients may be related to the acute phase reaction after 2019-nCoV infection. The increase in plasma FIB and other large molecule proteins can lead to an obvious increase in blood viscosity and hypercoagulable state, which facilitate the formation of thrombi in 2019-nCoV patients (10). Coagulation fibrinolytic system dysfunction may be related to inflammatory stimuli. Clinical studies have shown that elevated levels of FIB are related to infection severity and prognosis and that the FB level can be used as an indicator of the severity and prognosis of 2019-nCoV patients. (8, 11) DD is a specific molecular marker for cross-linked fibrin degradation. An increase in the plasma DD concentration indicates the formation of a floating thrombus in the body, which is commonly seen in various thrombotic diseases, such as deep vein thrombosis, pulmonary infarction, and disseminated intravascular coagulation (DIC) (12). Most of the patients reported in this paper had an increase in plasma DD, and the increase was quite significant. The elevation in plasma DD in most 2019-nCoV patients suggested the presence of hyperactivity of coagulation and fibrinolysis in the body. Related studies have shown that plasma DD levels can be used to predict 2019-nCoV pneumonia severity and prognosis.

High levels of D-dimer have been reported to be associated with 28-day mortality in patients diagnosed with infection or sepsis in the emergency department. One study demonstrated that DD levels higher than 1 mg/L, higher SOFA scores, and older age at admission were associated with a higher risk of in-hospital death. The mechanism includes systemic proinflammatory cytokine responses, which are mediators of atherosclerosis, directly leading to plaque rupture through local inflammation, induction of procoagulant factors, and hemodynamic changes, subsequently predisposing patients to ischemia and thrombosis (13).

Interleukin-6 (IL-6) is a cytokine that is secreted by immune cells. It is very important to the body's immune response and bone marrow hematopoietic and inflammatory reactions and is an important factor in maintaining the body's physiological balance. IL-6 not only has a high sensitivity and strong specificity inflammatory index but is also positively correlated with FIB and DD (14). A marked increase in IL-6 and other inflammatory cytokines during pneumonia was demonstrated in this study. The expression of IL-6 mRNA in buffy coat cells suggests that infiltrating leukocytes might contribute significantly to IL-6 levels detected in whole lung homogenates (15). High serum IL-6 levels are associated with the presence of pneumonia, disease severity, and poor prognosis. This suggests that inflammatory reactions are associated with blood coagulation fibrinolytic system disorders, and in relation to disease severity, they can be used for the diagnosis and prognosis of infectious diseases and reflect the severity of pulmonary infection in 2019-nCoV-infected patients (16). Whether patients with 2019-nCoV pneumonia need early anticoagulant therapy according to the related coagulation factors may improve their therapeutic effect and prognosis (17, 18).

Similarly, patients with Mycoplasma pneumoniae (MP) infection may have abnormal blood coagulation function, which can lead to embolism. Studies have found that some MP cases showed coagulation abnormalities (19). However, the relationship between embolization and MP infection is still not clear. Some studies have shown that the coagulation system is activated by the activation of the complement system caused by mycoplasma. Some authors concluded that mycoplasma infection and the produced cytokines cause damage to the vascular wall, leading to an imbalance in coagulation and anticoagulation factors and then to local vasculitis and thrombotic vascular occlusion. Some inflammatory factors, such as interleukin and tumor necrosis factor-α, accelerate coagulation. In fact, MP infection activates both exogenous and endogenous coagulation systems through a variety of pathways, leading to coagulation abnormalities and promoting thrombosis. Moreover, when liver cells are damaged by inflammation, the synthesis of several anticoagulation factors, including protein C and antithrombin III (AT-III), is affected, which also leads to the promotion of coagulation (20, 21).

In addition, many studies have proven that there are substantial coagulation abnormalities in pneumonia with severe sepsis, such as community-acquired pneumonia (22). Local activation of the coagulation system is known to occur in pneumonia, with fibrin deposition in the alveolar compartment to help contain infections, but it also enhances vascular permeability, stimulates proinflammatory cytokines, and promotes neutrophil accumulation. This local coagulation activation appears to be driven primarily by tissue factors. Normally, few tissue factors are exposed to circulating blood (23). However, alveolar macrophages, endothelial cells, and neutrophils can express tissue factors on their surfaces, which may produce a blood-borne pool of high thrombogenic tissue factors to promote the development of systemic coagulopathy during lung infection (24–26).

## Limitation

This study has several limitations. First, we included only 178 confirmed patients with COVID-19, all of whom had mild clinical symptoms. Suspected but unconfirmed cases were excluded from the analysis. It was better to include as many patients as possible in Wuhan or in other domestic cities in China or even abroad to obtain a more comprehensive understanding of the abnormal coagulation function of 2019-nCoV. Second, more detailed patient information, especially regarding clinical outcomes, was not available for analysis; however, the data from this study allowed for a better assessment of the clinical characteristics of COVID-19 coagulation function in Wuhan, China.

## Conclusion

In summary, in COVID-19 patients with mild clinical symptoms, regardless of whether they were hospitalized, all eight indicators were increased within 2 weeks after infection, and the three indicators, namely, DD, FIB, and IL-6, showed the most significant increases. After 2 weeks of infection, these indicators gradually decreased and returned to normal levels.

## Data Availability Statement

The original contributions presented in the study are included in the article/supplementary material, further inquiries can be directed to the corresponding author/s.

## Ethics Statement

This article does not contain any studies with animals performed by any of the authors. All procedures performed in studies involving human participants were in accordance with the ethical standards of the institutional and/or national research committee and with the 1964 Helsinki declaration and its later amendments or comparable ethical standards. The study was approved by the ethics committee of the Second Affiliated Hospital of Nanchang University. Informed consent was obtained from all individual participants included in the study.

## Author Contributions

JX: designed the research and drafting the manuscript. YZ: language polish and data analysis. YL: drafting the manuscript and literature search. KL: literature search and data analysis. XioZ, XiaZ, RM, KW, YG, FH, and JX: clinical data collection. WZ: clinical consultation and guidance. JQ: review and revise, guidance, and clinical data collection.

## Conflict of Interest

The authors declare that the research was conducted in the absence of any commercial or financial relationships that could be construed as a potential conflict of interest.
